# Supplementary figures and images for: Insular Celtic population structure and genomic footprints of migration
Source: PLoS Genet. 2018 Jan 25;14(1):e1007152. doi: 10.1371/journal.pgen.1007152 (PMC5784891; doi:10.1371/journal.pgen.1007152)

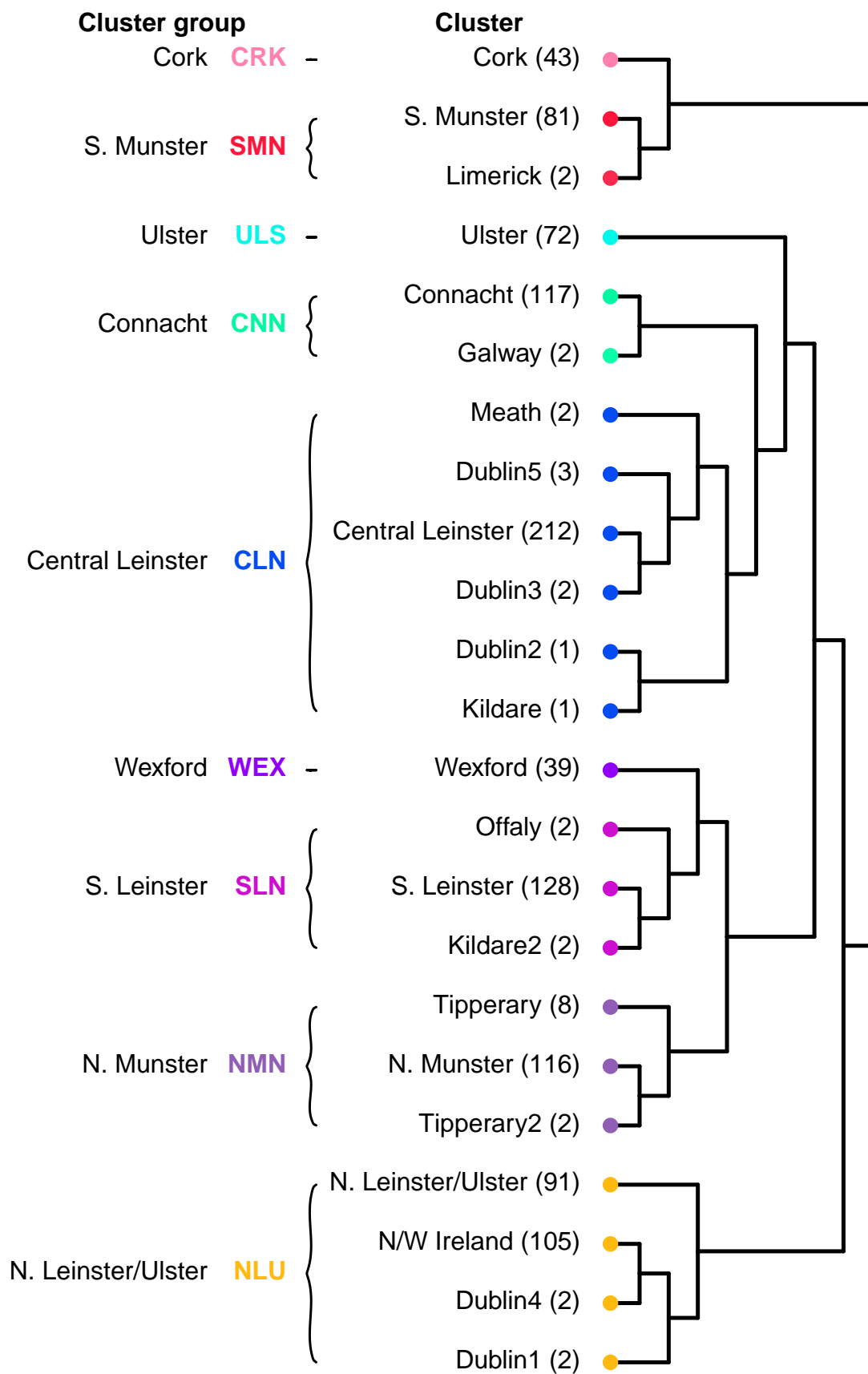

Supplement: S1 Fig — The fineSTRUCTURE tree presented in Fig 1 for Irish clusters with detailed breakdown of individual clusters. The individual labels for the clusters describe the geographic location of the majority of samples and the numbers of individuals within those clusters are provided in brackets. Cluster groups are identical to those defined in Fig 1. (PDF) [file pgen.1007152.s001.pdf]

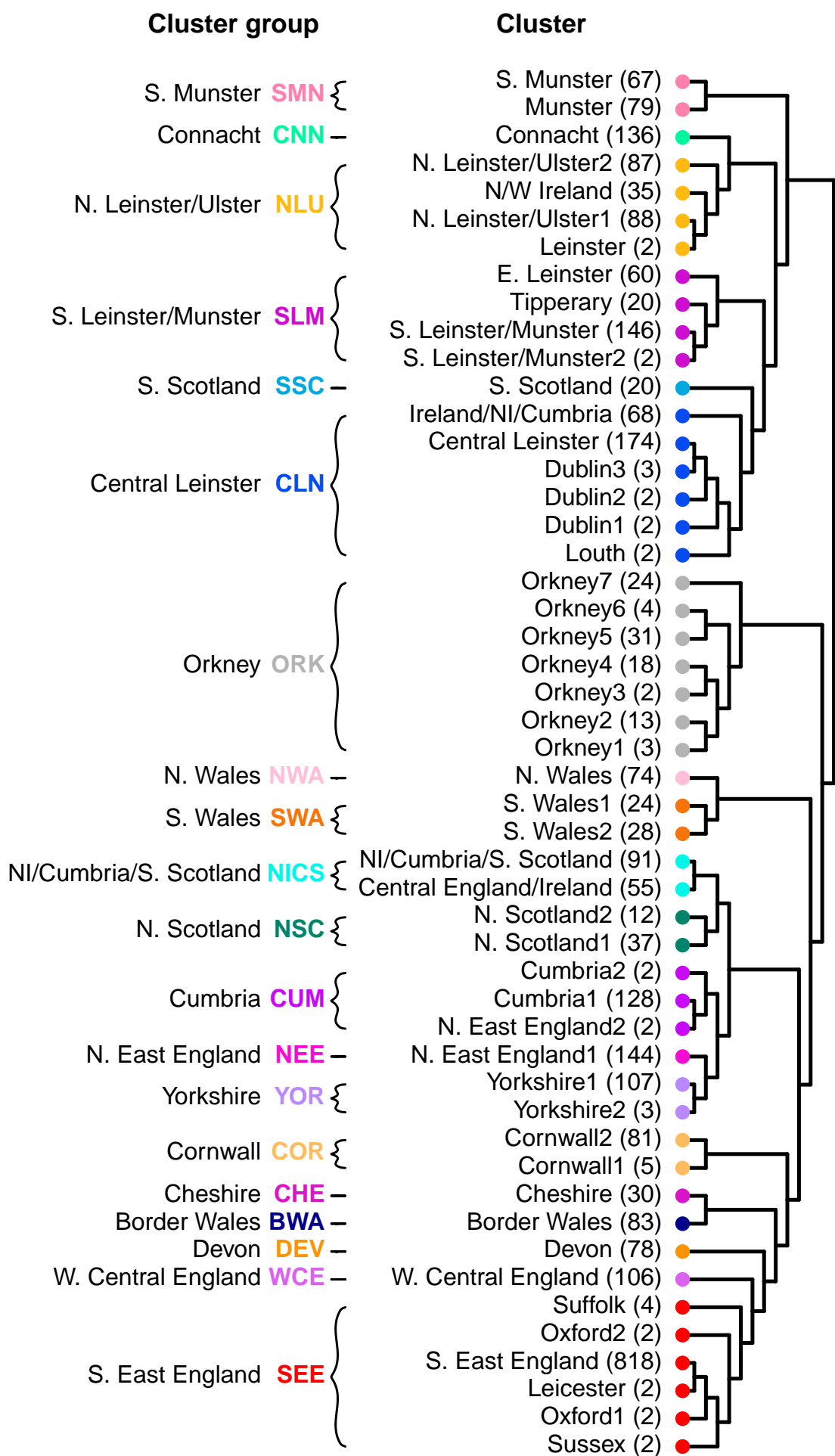

Supplement: S2 Fig — The fineSTRUCTURE tree presented in Fig 2 for British and Irish clusters with detailed breakdown of individual clusters. The individual labels for the clusters describe the geographic location of the majority of samples and the numbers of individuals within those clusters are provided in brackets. Cluster groups are identical to those defined in Fig 2. (PDF) [file pgen.1007152.s002.pdf]

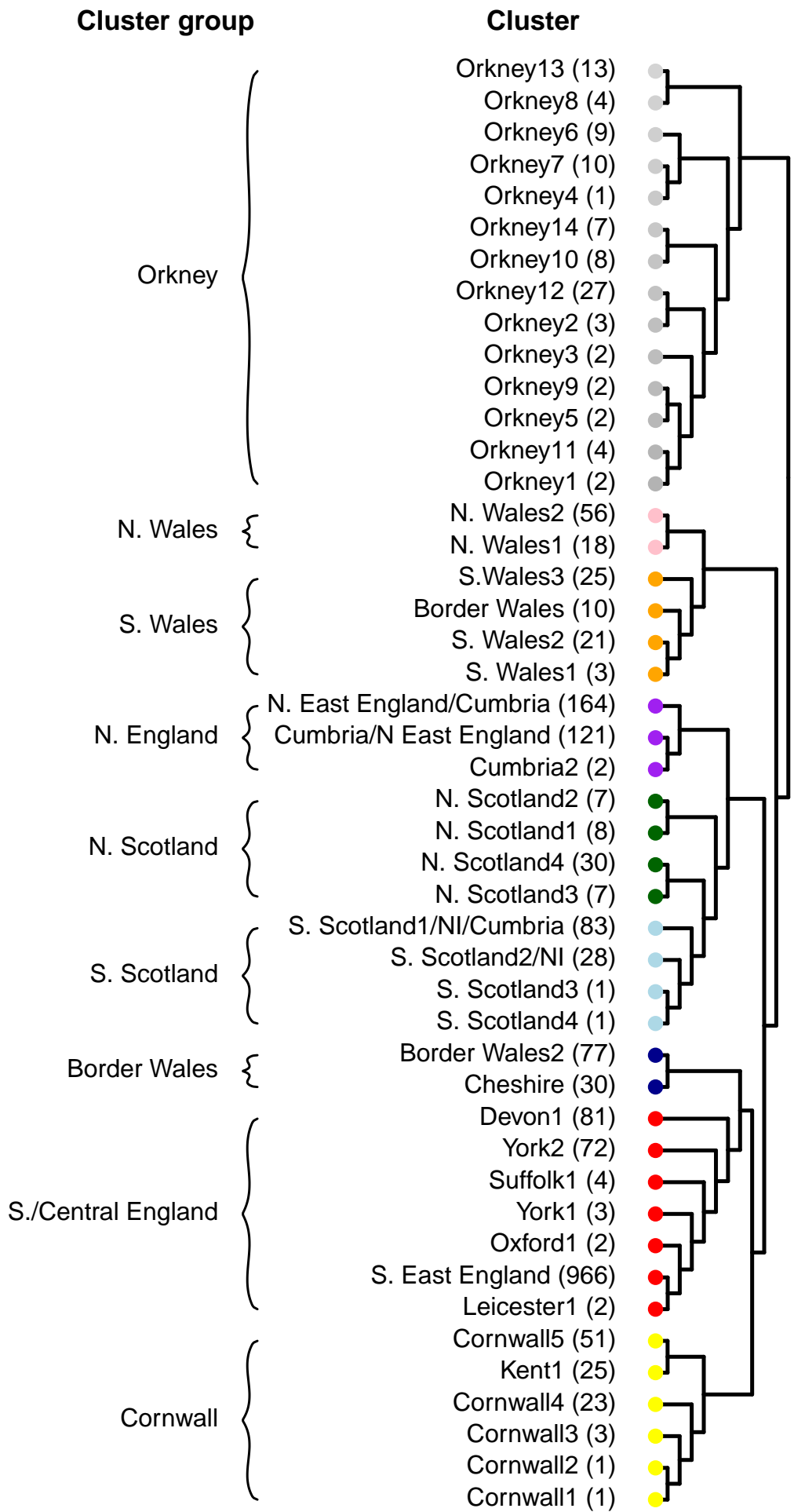

Supplement: S3 Fig — The fineSTRUCTURE maximum concordance tree for British clusters used in GLOBETROTTER analysis with detailed breakdown of individual clusters. The individual labels for the clusters describe the geographic location of the majority of samples and the numbers of individuals within those clusters are provided in brackets. Cluster groups describe clusters which are neighbouring in the tree and geographically adjacent. (PDF) [file pgen.1007152.s003.pdf]

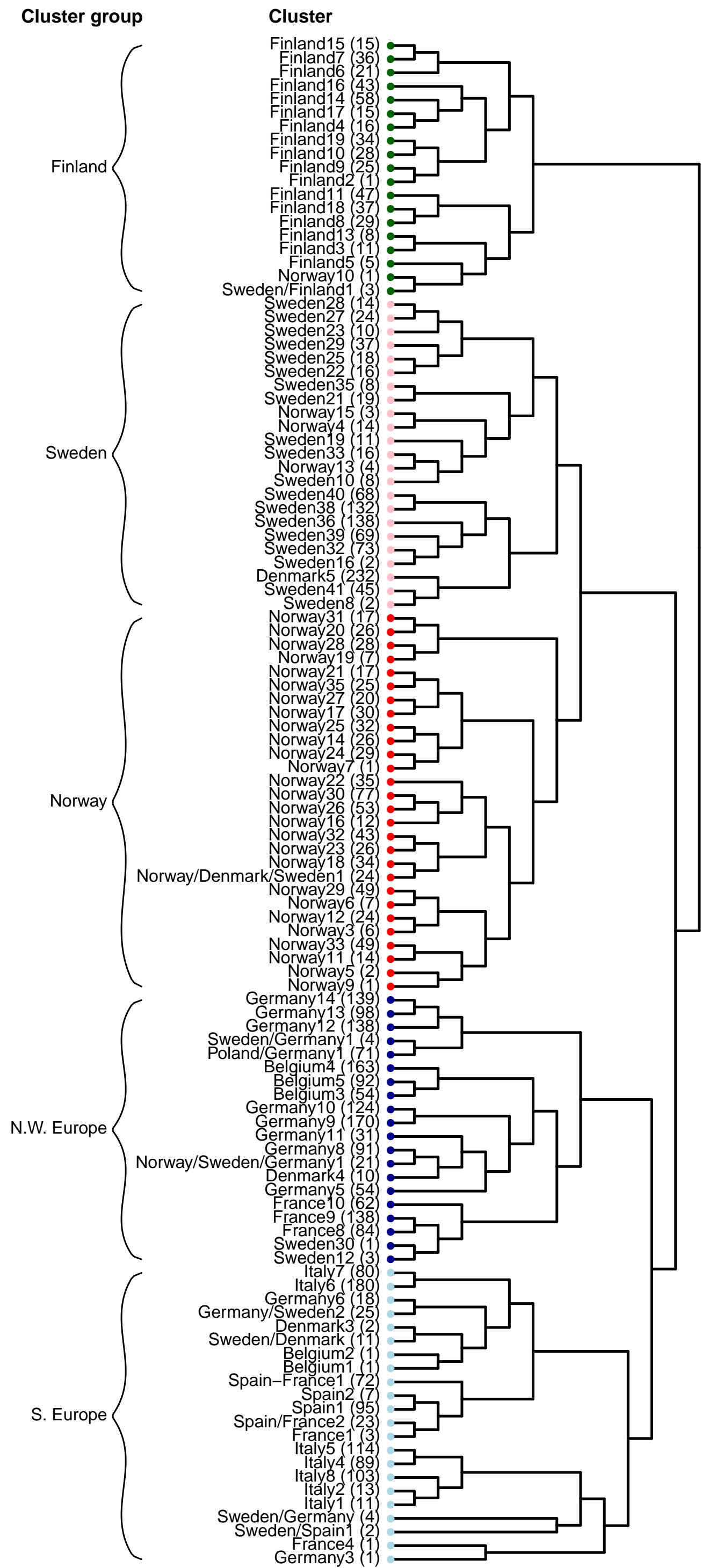

Supplement: S4 Fig — The fineSTRUCTURE maximum concordance tree for European clusters used in GLOBETROTTER analysis with detailed breakdown of individual clusters. Additional individuals from WTCCC exclusion list have been removed post fineSTRUCTURE clustering but prior to GLOBETROTTER analysis and the tree updated to reflect this. The individual labels for the clusters describe the geographic location of the majority of samples and the numbers of individuals within those clusters are provided in brackets. Cluster groups describe clusters which are neighbouring in the tree and geographically adjacent. (PDF) [file pgen.1007152.s004.pdf]

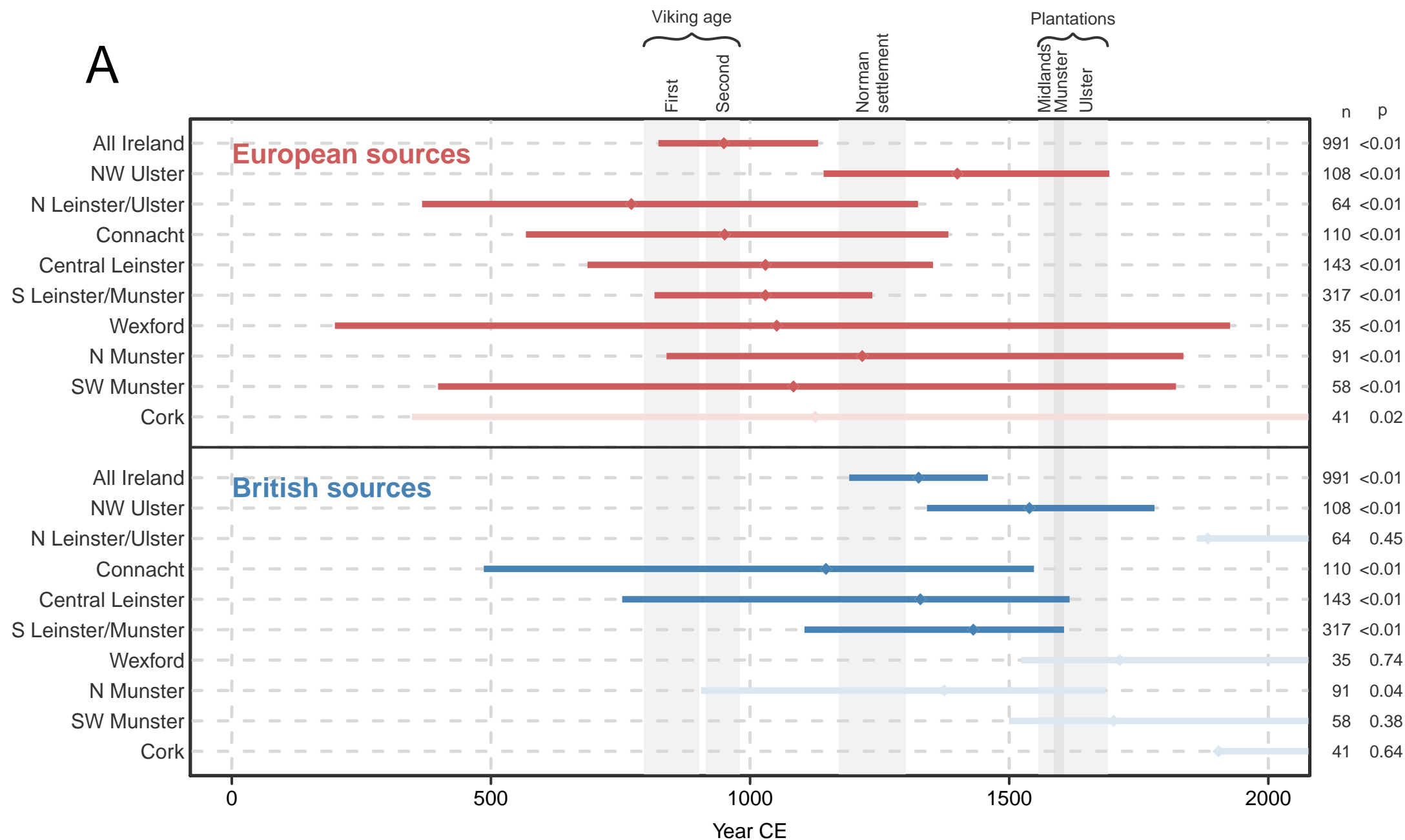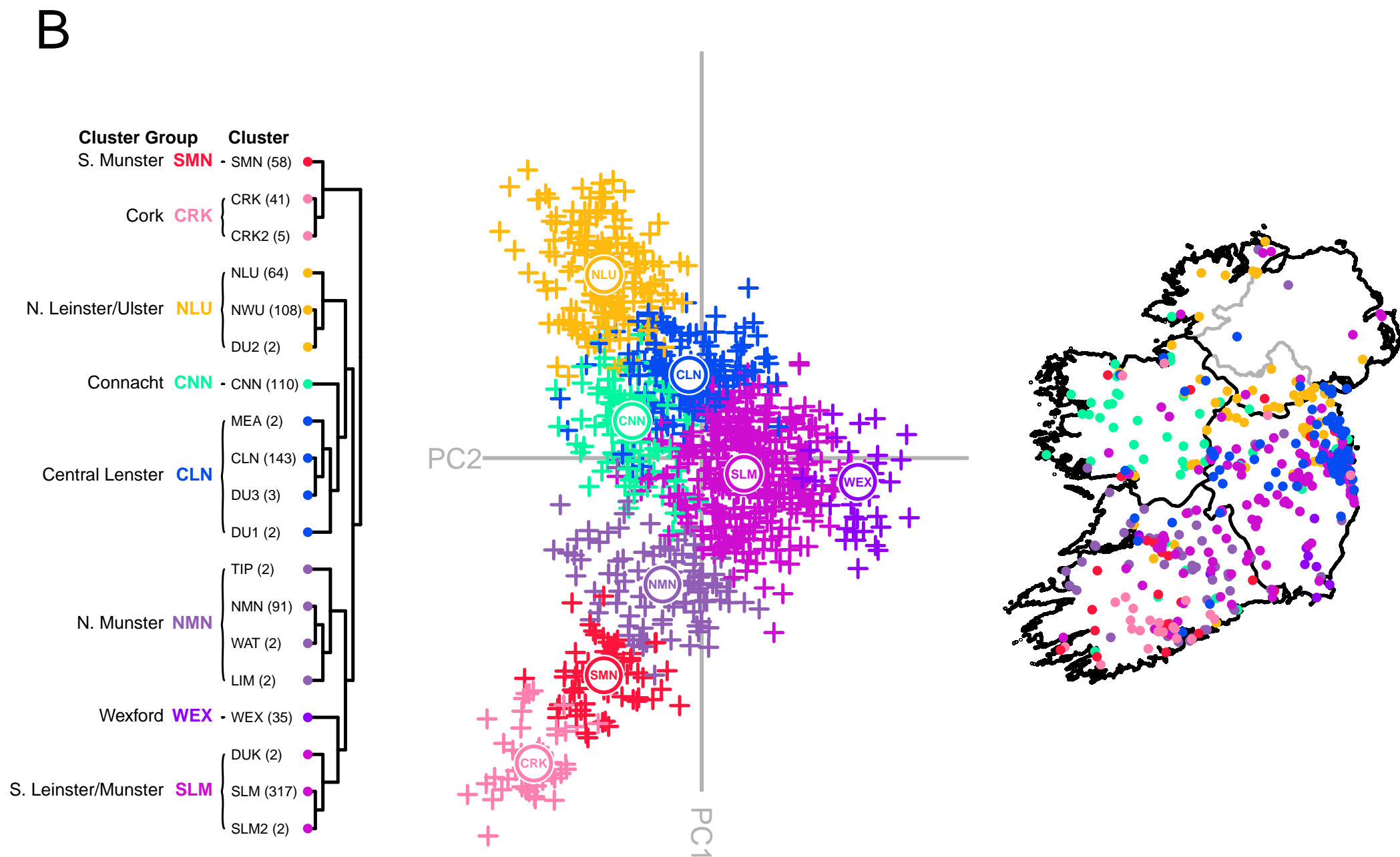

Supplement: S5 Fig — A summary of the date estimates and 95% confidence intervals for inferred admixture events into Irish clusters from European (red) and British (blue) admixing sources is shown in (A). Faded lines highlight clusters in which there was no significant evidence of admixture (P>0.01). (B) Summarises the fineSTRUCTURE maximum concordance tree cluster assignment for the 991 Irish samples used as target populations in GLOBETROTTER estimates in (A). We present the fineSTRUCTURE clustering dendrogram, a PCA of the coancestry matrix coloured by cluster group and a map of Ireland showing the sampling location for a subset of 544 individuals for which locational information was available, coloured by cluster group. Points have been randomly jittered within a radius of 5 km to preserve anonymity. The map and administrative boundaries were produced using data from the database of Global Administrative Areas (GADM; https://gadm.org). (PDF) [file pgen.1007152.s005.pdf]

K=2

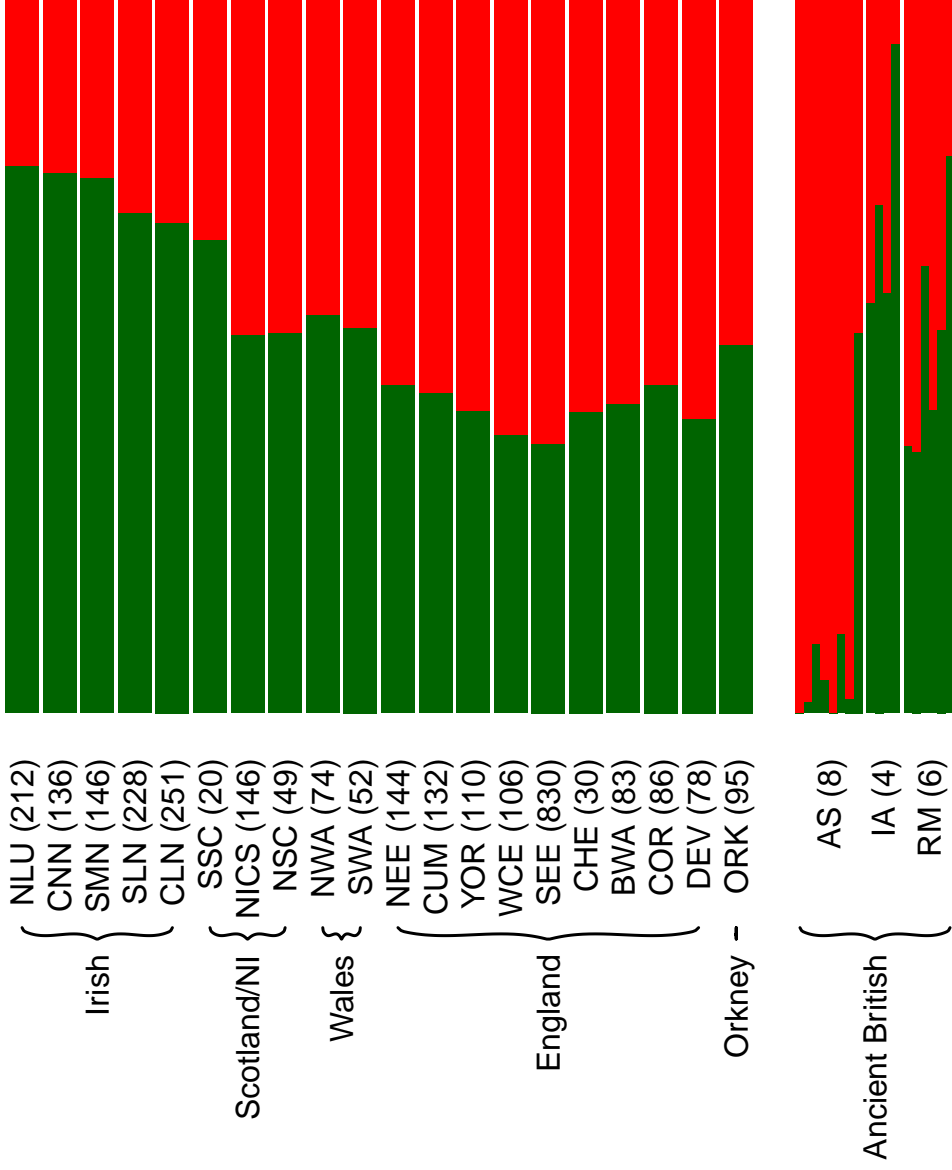

Supplement: S8 Fig — ADMIXTURE component (k = 2) for each cluster group in the PoBI/Irish fineSTRUCTURE tree (S2 Fig) and 18 Ancient British Samples from the Iron age (IA; n = 4), Anglo-Saxon (AS; n = 8) and Roman (RM; n = 6) periods. Admixture proportions are averaged across each cluster group (left) for brevity of display, while individual proportions are plotted for ancient samples. The Anglo-Saxon individuals are best described by the red component. This component is high in British cluster groups from areas affected by the Anglo-Saxon invasion such as the large SEE cluster, while relatively low in Celtic populations such as Ireland, Scotland and Wales. (PDF) [file pgen.1007152.s008.pdf]

**A**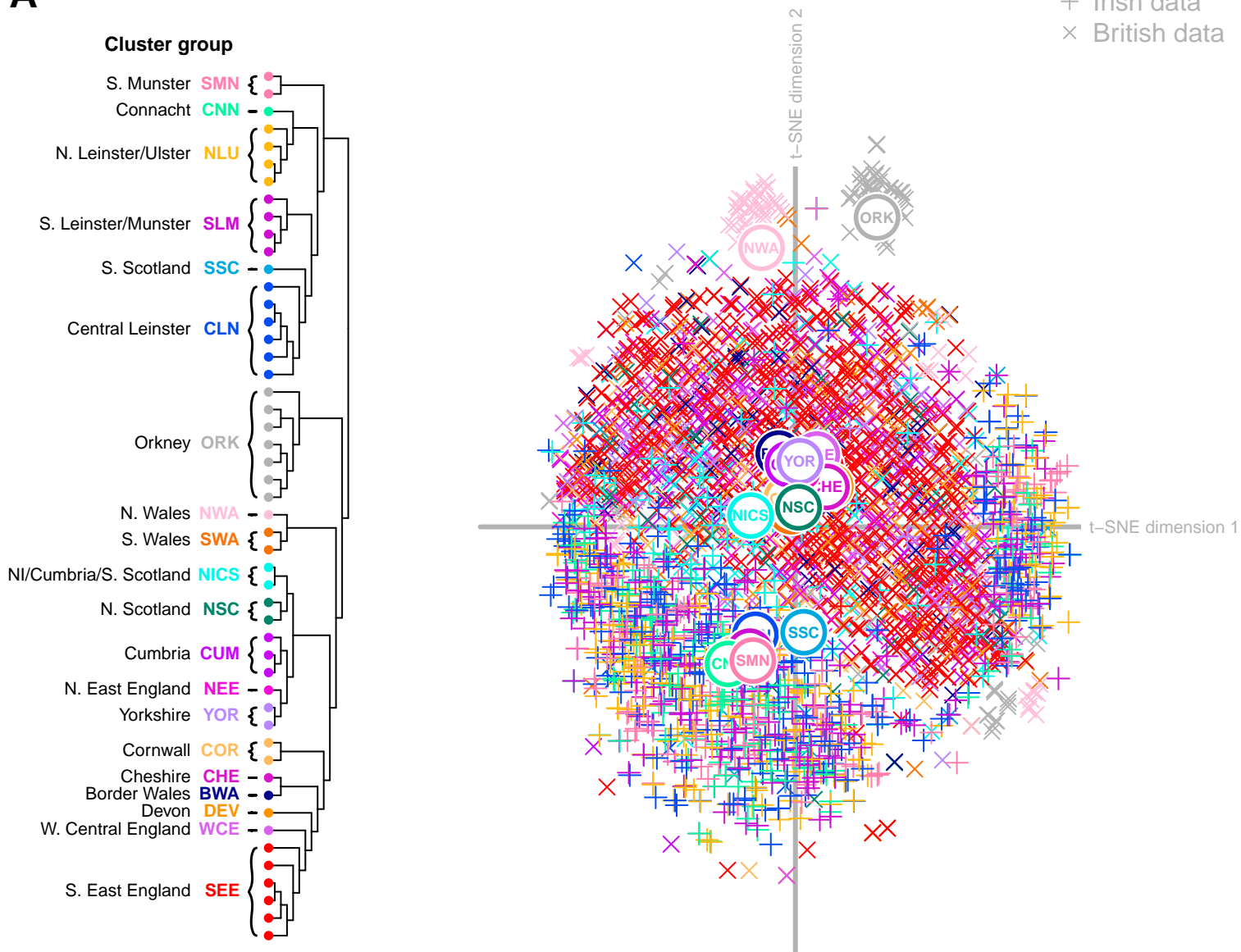

Supplement: S9 Fig — A t-SNE solution for 2-dimensional embedding is displayed for Irish and British genotype data using Hamming distances (identity-by-state). As t-SNE is a stochastic method, different runs produce different solutions to the 2-dimensional embedding; shown here is a typical result. Clusters and cluster groups are defined as in Fig 2, with median locations for cluster groups plotted. t-SNE performed significantly worse with the Hamming distances (identity-by-state) computed over single SNP markers than with the fineSTRUCTURE coancestry matrix (Fig 3). (PDF) [file pgen.1007152.s009.pdf]

British component

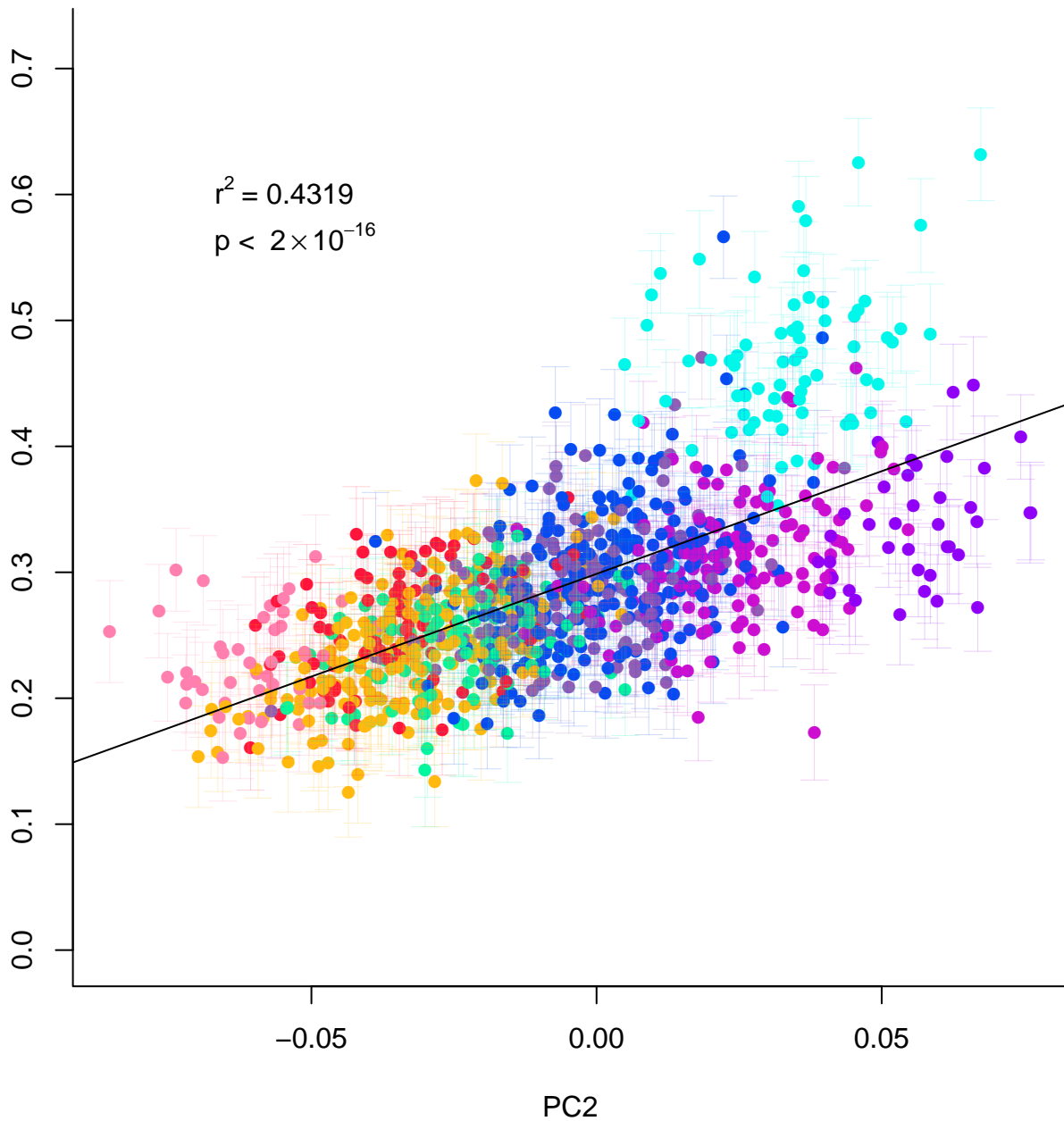

Supplement: S11 Fig — Standard error calculated using 200 bootstrap resamples for each point in linear regression in Fig 1 (E.) are plotted using error bars to show variability in ADMIXTURE point estimates. (PDF) [file pgen.1007152.s011.pdf]
