# Supplementary material for: Insular Celtic population structure and genomic footprints of migration
Source: PLoS Genet. 2018 Jan 25;14(1):e1007152. doi: 10.1371/journal.pgen.1007152 (PMC5784891; doi:10.1371/journal.pgen.1007152)
Supplement: S6 Fig — Bar charts displaying the GLOBETROTTER estimated British ancestry profile for Republic of Ireland clusters (Defined in S5 Fig; Only clusters with 35+ samples displayed) from British clusters inferred from 2,017 individuals using fineSTRUCTURE (Defined in S3 Fig). Individuals from Northern Ireland were excluded to prevent masking of ancestry leaving 1973 individuals. Only donors that make at least a 2.5% contribution to at least one Irish cluster are displayed with the remaining proportions subsumed into the “other” category. Error bars represent the bootstrapping procedure with 10000 resamples (Black) and a jack-knife approach using 22 resamples (Red). Label abbreviations: S_SCOT, south Scotland; SE_ENG, southeast England; CHE, Cheshire; KEN, Kent; BWA, border Wales; DEV, Devon; COR, Cornwall; N_SCOT north Scotland; SWA, south Wales; NWA, north Wales. (PDF) [file pgen.1007152.s006.pdf]

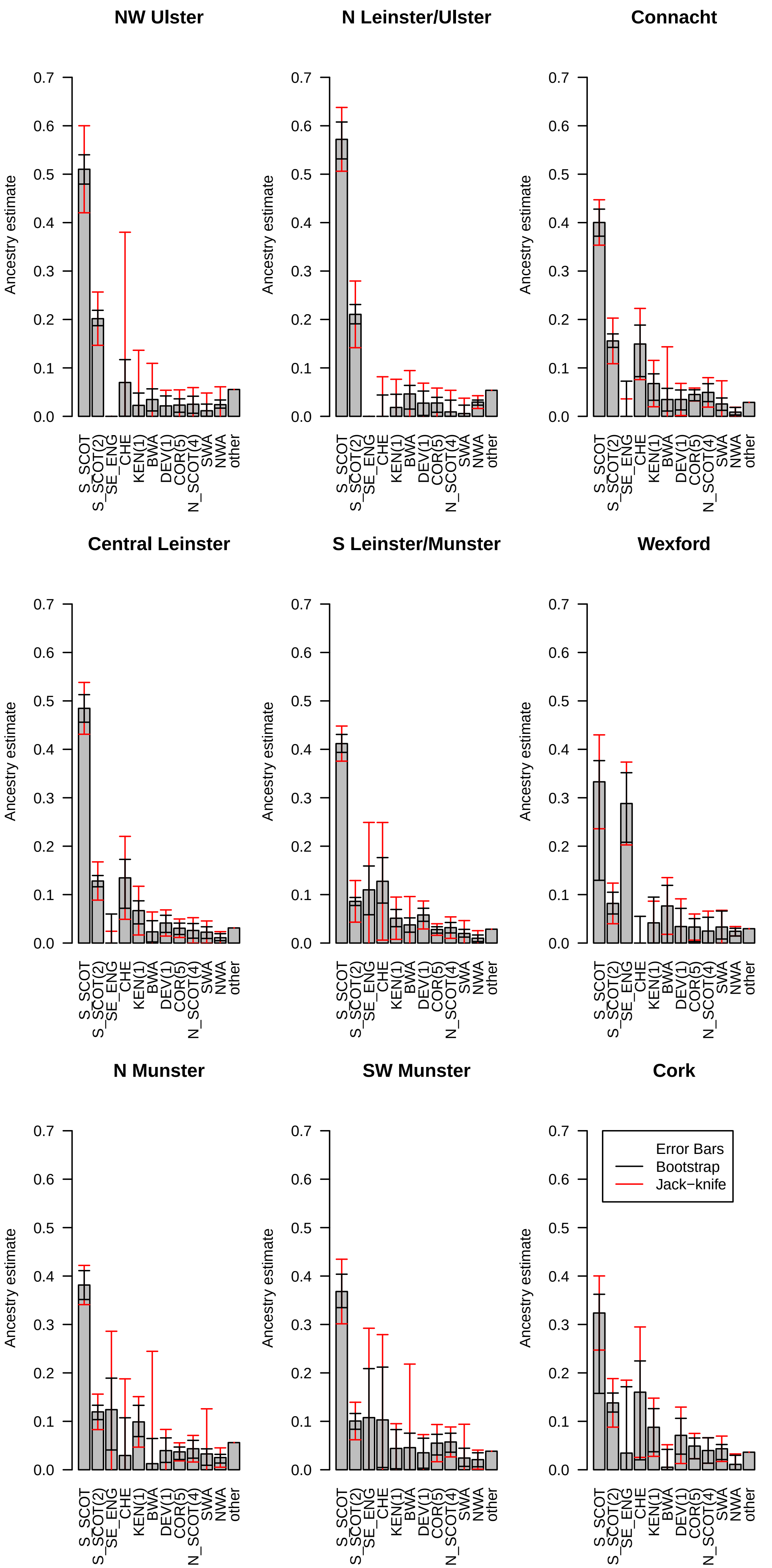

NW Ulster
N Leinster/Ulster
Connacht
Central Leinster
S Leinster/Munster
Wexford
N Munster
SW Munster
Cork
Ancestry estimate
0.0
0.1
0.2
0.3
0.4
0.5
0.6
0.7
S_SCOT
S_SCOT(2)
SE_ENG
CHE
KEN(1)
BWA
DEV(1)
COR(5)
N_SCOT(4)
SWA
NWA
other
Error Bars
Bootstrap
Jack−knife
