# Supplementary material for: Insular Celtic population structure and genomic footprints of migration
Source: PLoS Genet. 2018 Jan 25;14(1):e1007152. doi: 10.1371/journal.pgen.1007152 (PMC5784891; doi:10.1371/journal.pgen.1007152)
Supplement: S7 Fig — Bar charts displaying the GLOBETROTTER estimated European ancestry profile for republic of Ireland clusters (Defined in S5 Fig; Only samples with 35+ samples displayed) from European clusters inferred from 4,514 individuals using fineSTRUCTURE (Defined in S4 Fig). Only donors that make at least a 2.5% contribution to at least one Irish cluster are displayed with the remaining proportions subsumed into the “other” category. Error bars represent the bootstrapping procedure with 10000 resamples (Black) and a jack-knife approach using 22 resamples (Red). Label abbreviations: NOR-SG, Norway, with significant minor representations from Sweden and Germany; FRA, France; NOR, Norway; BEL, Belgium. (PDF) [file pgen.1007152.s007.pdf]

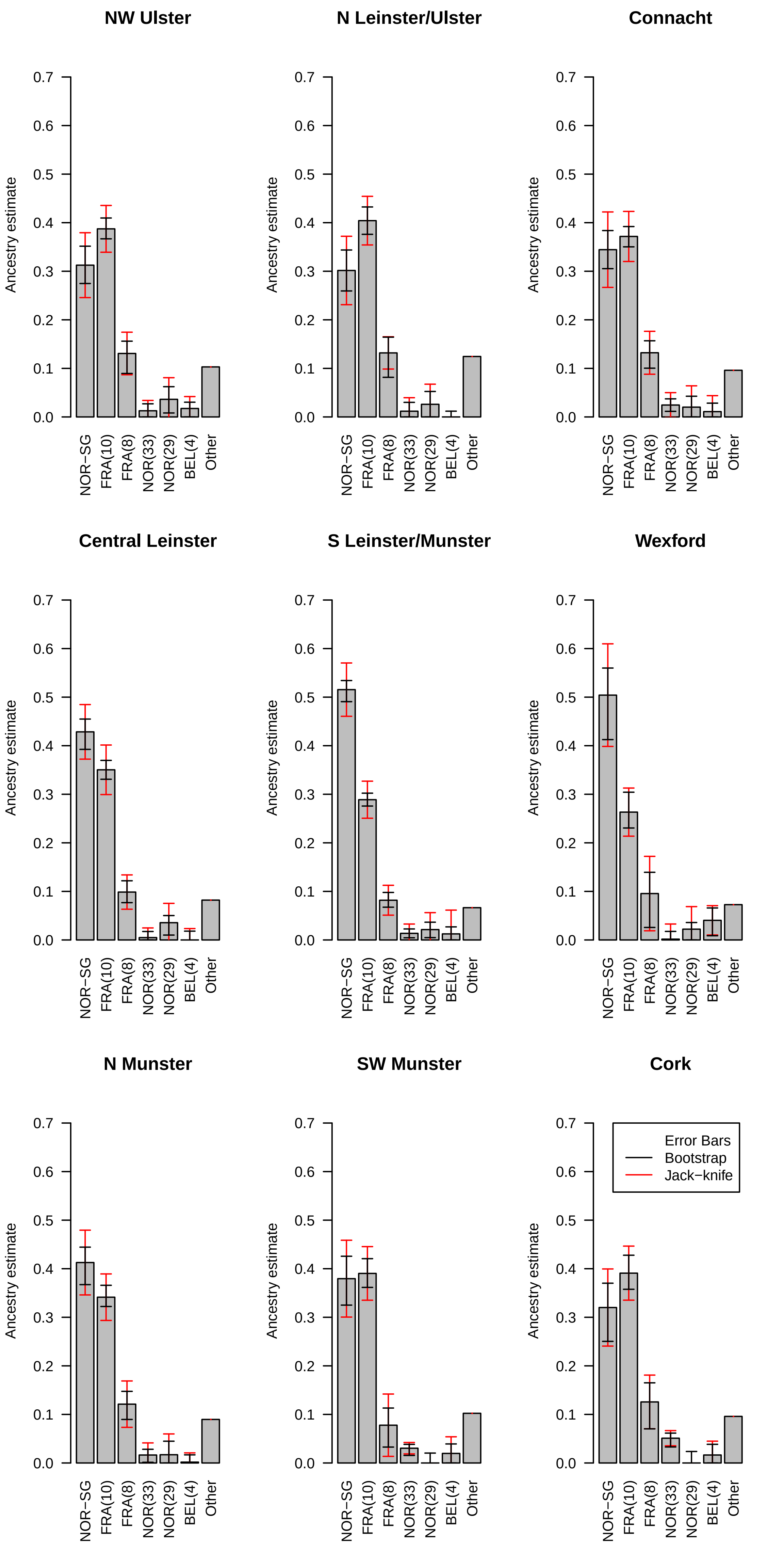
NW Ulster
N Leinster/Ulster
Connacht
Central Leinster
S Leinster/Munster
Wexford
N Munster
SW Munster
Cork
Ancestry estimate
0.0
0.1
0.2
0.3
0.4
0.5
0.6
0.7
NOR−SG
FRA(10)
FRA(8)
NOR(33)
NOR(29)
BEL(4)
Other
Error Bars
Bootstrap
Jack−knife
