# Supplementary material for: Insular Celtic population structure and genomic footprints of migration
Source: PLoS Genet. 2018 Jan 25;14(1):e1007152. doi: 10.1371/journal.pgen.1007152 (PMC5784891; doi:10.1371/journal.pgen.1007152)
Supplement: S10 Fig — Displays ChromoPainter PC1 and PC2 alongside a fineSTRUCTURE Maximum Concordance clustering dendrogram for A.) Linked and B.) Unlinked analysis for 991 Irish individuals at the 166,139 SNP positions used for our European GLOBETROTTER run. Trees and PCA are coloured at a k = 11 split for ease of visualisation. Considerably more structure is apparent in the PCA of the Linked analysis indicating that linkage information defines meaningful haplotypes even at this resolution. We report “Confidence of ind. assignment” for each method. This metric is the confidence of individual assignment to their final cluster based on their assignment across all MCMC samples defined in PoBI [7]. This was on average 84.8% (95% CI: 83.9–85.7%) for the Linked analysis, while in the Unlinked analysis this was only 8.06% (95% CI: 8.03–8.09%), suggesting that the final clustering assignment in the unlinked mode is extremely uncertain and variable. (PDF) [file pgen.1007152.s010.pdf]

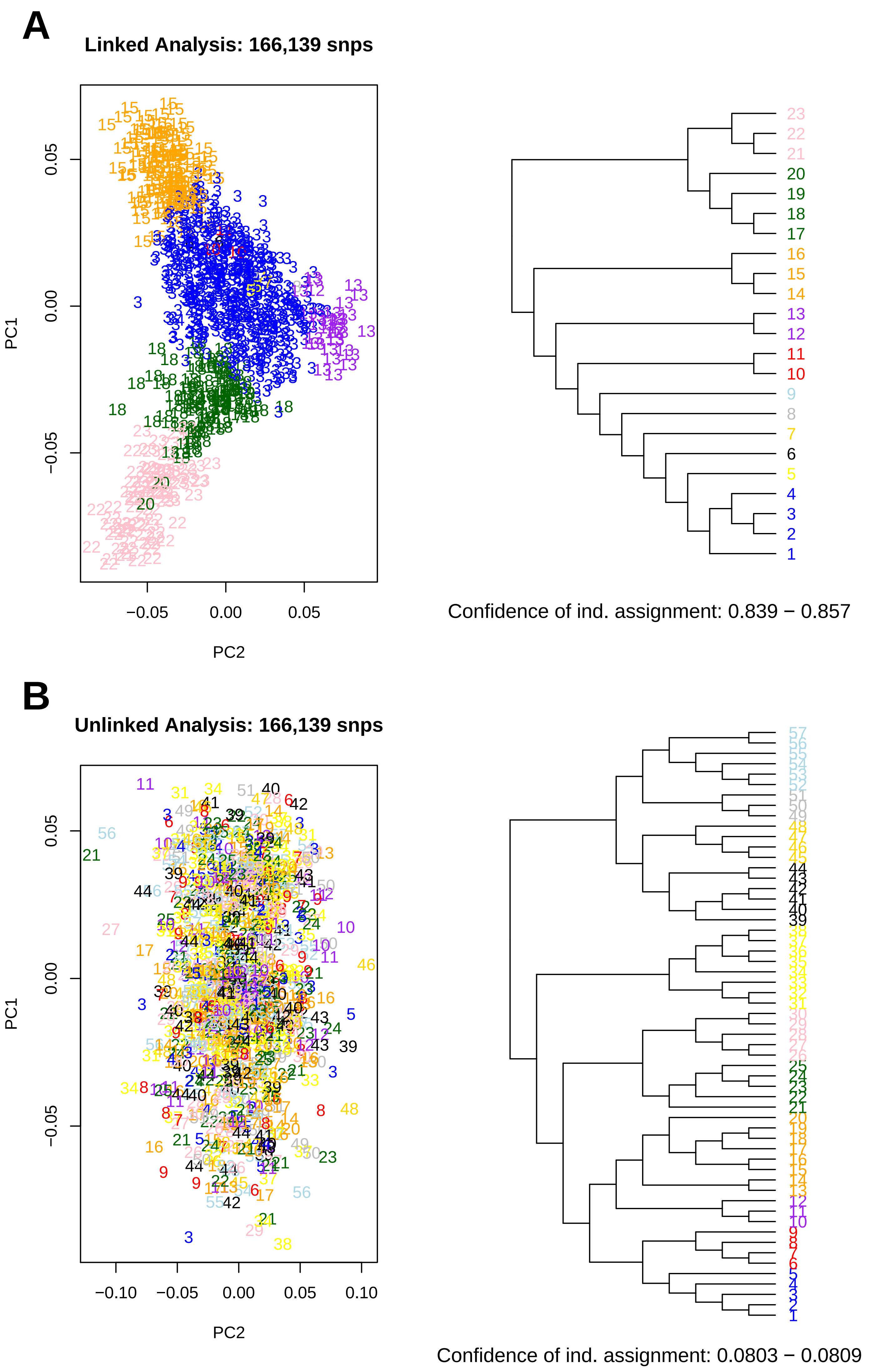
A
Linked Analysis: 166,139 snps
PC1
PC2
Confidence of ind. assignment: 0.839 − 0.857
B
Unlinked Analysis: 166,139 snps
PC1
PC2
Confidence of ind. assignment: 0.0803 − 0.0809
