# Supplementary material for: Insular Celtic population structure and genomic footprints of migration
Source: PLoS Genet. 2018 Jan 25;14(1):e1007152. doi: 10.1371/journal.pgen.1007152 (PMC5784891; doi:10.1371/journal.pgen.1007152)
Supplement: S1 Text — (PDF) [file pgen.1007152.s019.pdf]

S1 - Supplementary Text : Supplementary Methods

# 1.) Populations

We utilised three distinct population-scale datasets in our study: a newly generated Irish dataset (n = 1,120) sampled from the Irish ALS DNA bank (536 cases; 584 controls; see Irish Samples), the people of the British Isles dataset (n = 2039) [1] and European MS dataset (n = 6,760) [2]. Merges of the Irish data with the British and European datasets were used to explore the broader genetic context of Ireland.

### Irish Samples (IRE)

407 Irish samples (271 cases; 136 controls) from the Irish ALS DNA bank were genotyped at 2.5 million single nucleotide polymorphisms (SNPs) using the Infinium HumanOmni2.5-8 SNP array v1.2. This dataset was merged with Irish data from a published dataset [3] containing 713 individuals (265 cases; 448 controls) genotyped on the Infinium omni express-24 kit. The final merge of these datasets following quality control (QC) contained a total 991 Irish individuals and 407,750 SNPs.

### UK samples (UK)

From an initial 2,039 geocoded samples 2,020 individuals passed QC, genotyped at a total of 521,833 SNP sites. These were included in the initial UK-only fineSTRUCTURE run to define homogenous clusters for use as surrogate/donor populations in GLOBETROTTER.

### European Samples (EUR)

Following exclusion of non-European individuals from the MS dataset we had 6,670 individuals. Our initial QC reduced the European dataset to 4,737 individuals genotyped at 363,396 SNPs. Further removal of 223 individuals recommended by the WTCCC quality control left 4,514 individuals.

### UK-Irish Merge (UK-IRE)

Following merge of Irish and UK datasets and an additional round of QC, three additional UK individuals were removed. The final UK-Irish merge contained 3,008 individuals and 214,632 SNPs. This dataset was used for the Ireland/Britain fineSTRUCTURE analysis and the GLOBETROTTER analysis.

We also used a subset of this dataset containing Irish samples (n=991) and individuals from Northern Ireland from the UK dataset (n=44) amounting to a complete dataset of 1,035 individuals from the island of Ireland genotyped at 214,632 SNPs

### European-Irish merge (EUR-IRE)

Following merge and additional QC our European-irish merge contained 5,506 individuals at 166,139 SNPs.

## 2.) Quality control

We applied the following quality control for samples and SNPs to each dataset individually using PLINK 1.9 [4]. Uncommon SNPs with a minor allele frequency less than 5% were filtered (--maf 0.05), as were SNPs with a missingness across samples greater than 2% (--geno 0.02). Individuals with a high overall missingness of remaining SNPs (--mind 0.1) or excess heterozygosity (--het) greater than 3 median absolute deviations from the median were removed. An identity-by-descent matrix was calculated between pairs of individuals (--genome) and the individual with higher missingness among pairs with greater than 12.5% relatedness (pi-hat > 0.125) was removed from downstream analysis. Principal component analysis (PLINK --pca) was used to detect and remove population outliers in the Irish population (>4 standard deviations from mean for principal components 1-2) for the IRE and EUR-IRE datasets. Finally all SNPs with any missingness were removed (--geno 0) prior to running ChromoPainter.

We noted the SNP missingness filter (--geno 0) led to a significant loss of SNPs in the EUR dataset, particularly after merging due to poor overlap of SNP panels with the IRE dataset. Given that the dataset was solely intended for use in the merge with the IRE dataset, we resolved to balance this SNP loss by removing individuals from the analysis above a threshold of missingness prior to applying the --geno 0 filter. We assayed a range of thresholds to maximise SNP count while minimising individual loss using the --mind command in PLINK. We settled on the threshold --mind 0.0005 which maintained sufficient SNPs and individuals for a meaningful analysis (see S1 Populations).

As the EUR dataset is composed of multiple sclerosis patients and controls we took the precaution of removing any SNPs in a 15 Mb region surrounding the strongly associated HLA locus (chr6:22,915,594-37,945,593), following previous studies [1,5], to avoid haplotypic bias arising from this association. This was also done for all merged datasets containing the EUR data.

Finally, following preliminary fineSTRUCTURE clustering of Europeans to define homogeneous groups, any remaining European samples which failed the WTCCC2 QC were removed to ensure that only high quality and thus representative European samples were used in the GLOBETROTTER analysis (S4 GLOBETROTTER).

A pre-phasing QC step was also carried out (--check) using SHAPEIT v2 [6] to remove any SNPs which did not align well to the 1000 Genomes Project reference panel (see Phasing).

## 3.) fineSTRUCTURE analysis

We performed fineSTRUCTURE analysis as described in Lawson *et al.* [7] on each of the population datasets (IRE, UK, EUR) individually, and then separately on the merge of the IRE-UK merge.

For this analysis we initially modelled haplotype sharing between individuals using ChromoPainter [7] to generate a coancestry matrix, which records the expected number of haplotype chunks each individual donates to each other. Next we clustered individuals into ancestrally homogeneous sub-groups based on these patterns of haplotype sharing in this coancestry matrix using the fineSTRUCTURE algorithm.

For the ChromoPainter step we converted phased haps files to chromopainter phase files using the impute2chromopainter.pl script provided at http://www.paintmychromosomes.com and generated recombination maps using convertrecfile.pl. Using the version of ChromoPainter built into fineSTRUCTURE (fs-2.0.8) we performed 10 expectation maximisation iterations to estimate the parameters Ne and Mu (switch rate and mutation rate) for each chromosome (for the analysis in the IRE dataset, which contained data from Northern Ireland and Ireland, we used the weighted average for Ne and Mu across chromosomes 1,8,15,20). The model was then run for each chromosome using the average values for these parameters across all individuals. We used default settings to paint all individuals by all others (-a 0 0) with the exception of the k value which was set to 50 for all analyses including Irish or British data. This followed the PoBI study, which made the observation that Irish and British individuals often share slightly longer haplotypes than average [1]. We used the built in chromocombine script to combine all individual paintings across all chromosomes into a single coancestry matrix with a single normalisation parameter c to be used in the fineSTRUCTURE clustering.

We ran fineSTRUCTURE's MCMC model on the coancestry matrix for 2,000,000 burn-in and 2,000,000 sampling iterations, sampling every 10,000 iterations to determine the grouping of samples with the best posterior probability. For the larger EUR dataset this was

reduced to 1,000,000 burn-in and 1,000,000 sampling iterations, sampling every 1,000 iterations. The fineSTRUCTURE algorithm attempts to merge and split groups based on their haplotype sharing profile to achieve the best set of clusters. Two MCMC chains were run to allow for assessment of convergence on final cluster membership. We then extracted the state with the maximum posterior probability among all samples and performed an additional 10,000 hillclimbing iterations before inferring trees using either the default tree building method or the maximum concordance method described in the PoBI study [1].

The default tree building method samples possible merges and splits and selects the tree with the best posterior probability among sampled trees and hence does not consider the assignment of individuals but rather the total state of the final tree. The alternative maximum concordance method takes into account cluster membership across all MCMC samples to reassign each individual to their ideal cluster. The default tree method generally achieves greater splitting of the data and hence has the potential to reveal more structure, while having the potential to over-split. Conversely the maximum concordance tree returns fewer clusters with greater certainty in cluster assignment of individuals while having the potential to under-split.

We present a tree built with the default method for the IRE dataset in Fig 1 as both trees agreed well on a higher level and the splits determined by the default tree method are easier to interpret due to the lack of a reassignment step and the more intuitive merge/split algorithm used to build it. For Fig 2 (IRE-UK dataset) we present the maximum concordance tree as it is preferable to minimise spurious cluster assignment for individuals from genetically similar sources including Ireland and Scotland. We also present a tree cut at k=50 for this Irish/UK (although there were initially 62 clusters) to facilitate interpretation of the salient genetic structure of the British Isles. Finally, as the goal of fineSTRUCTURE in the GLOBETROTTER [8] analysis was to reduce confounding from inhomogeneous donor groups and not to detect potentially interesting structure, we favoured the conservative split given by the maximum concordance method to ensure the most homogenous clusters possible in European and British donor and recipient populations.

fineSTRUCTURE trees were visualised using R scripts provided at http//:www.paintmychromosomes.com and clusters identified were used to label the principal component analysis of the coancestry matrix. Cluster names were devised based on the geographic spread of a given cluster. This was based on reported sampling region for the PoBI and MS data. For Irish data we based this on geocoded locations of our samples.

## 4.) Mapping samples

Of the 991 Irish samples in this dataset after QC, geographic information was available for 544 in the form of home address. For the purposes of preserving anonymity this was jittered in all maps containing patients (Fig 1 and S5 Fig). Additionally, 44 PoBI individuals from Northern Ireland were used, but precise sampling location was not available so these are plotted as a circle in Fig 1. The map for Fig 1 was generated using Global Administrative areas from GADM version 2.8 (November 2015; http://www.gadm.org).

For UK data sample location was described in terms of membership of 35 sampling regions in supplement to the PoBI data [1]. To plot these regions in Fig 2 and Fig 3 we used the UK map and administrative boundary data from GADM (http://www.gadm.org) to approximate regions defined in NUTS 2010 [9] (Nomenclature of Territorial units for statistics). We then combined sets of these NUTS 2010 regions to best approximate the 35 sampling regions. The 35 sampling regions were then allotted to the cluster group containing the majority of samples from the respective sampling region and labeled accordingly. Where a cluster or cluster group had the majority of samples for multiple adjacent sampling regions (as in the case of southeast England), we subsumed these regions.

The Irish map in Fig 2 and Fig 3 was also plotted using data from GADM (http://www.gadm.org). For consistency with the UK map we divided Ireland into regions using the NUTS 2010 definitions [9], with each region assigned to the cluster with the majority of samples in that region as above.

## 5.) GLOBETROTTER analysis of admixture dates

We used the GLOBETROTTER method [8] to investigate signals of admixture into Ireland from Europe and Britain separately. GLOBETROTTER utilises ChromoPainter output to generate a mixture model for a target population's haplotypic makeup as a combination of donor populations, which can be thought of as surrogates for the true ancestral groups. The better the donor populations represent the admixing sources, the cleaner this model will fit and the more interpretable the results will be. Once a suitable reduced model is generated, GLOBETROTTER estimates the pairwise likelihood of being painted by two donor populations at a variety of genetic distances to generate coancestry curves. These curves can be used to date an admixture event based on a model of the exponential decay of linkage disequilibrium in admixed regions [8]. For a simple admixture event the rate of decay of the exponential will equal the number of generations since the event.

We used ChromoPainter v2 to paint target both Irish clusters (Irish clusters in S5 Fig) and surrogate populations with the following homogeneous donor populations which were defined with fineSTRUCTURE (Clusters defined in S3 Fig and S4 Fig; also used as surrogates):

a.) European clusters in the EUR dataset;

b.) British clusters from the UK dataset.

We estimated switch rate and mutation rate (Ne and Mu) for each analysis using 10 expectation maximisation iterations and taking the weighted average across 4 chromosomes (1, 8, 15 and 20) as recommended in the GLOBETROTTER manual. These parameters were used with standard settings to generate copy vectors for both target and surrogate populations, as well as 10 painting samples for each Irish individual. Note that copy vectors here represent the average chunk length copied rather than chunk count as this is more readily comparable and standardised across all individuals.

Globetrotter was run for five mixing iterations (num.mixing.iterations: 5) for each target population, both standardising against a null (null.ind: 1) and standard (null.ind: 0) to test for signals of admixture. We bootstrapped for admixture date 100 times and calculated the probability of a null model of no admixture by calculating the proportion of non-sensical inferred dates (<1 or >400 generations) produced by the model (null.ind: 1) as in the GLOBETROTTER paper. Confidence intervals for the date were calculated from the bootstraps for the standard model (null.ind: 0) using the empirical bootstrap method.

For the reported British analysis PoBI samples from Northern Ireland were excluded as donors from their respective clusters to prevent masking of admixture signals from other UK clusters given the expected constant gene flow between the Republic of Ireland and Northern Ireland leaving 1,973 British donors for consideration. Notably inclusion of Northern Irish individuals increased the confidence of the model however, perhaps as they are the cleanest proxy for the Plantation signal.

References:

1. Leslie S, Winney B, Hellenthal G, Davison D, Boumertit A, Day T, et al. The fine scale genetic structure of the British population. Nature. 2015;519: 309–314.

2. Sawcer S, Hellenthal G, Pirinen M, Spencer CCA, Patsopoulos NA, Moutsianas L, et al. Genetic risk and a primary role for cell-mediated immune mechanisms in multiple sclerosis. Nature. 2011;476: 214–219.

3. van Rheenen W, Shatunov A, Dekker AM, McLaughlin RL, Diekstra FP, Pulit SL, et al. Genome-wide association analyses identify new risk variants and the genetic architecture of amyotrophic lateral sclerosis. Nat Genet. 2016;48: 1043–1048.

4. Chang CC, Chow CC, Tellier LC, Vattikuti S, Purcell SM, Lee JJ. Second-generation PLINK: rising to the challenge of larger and richer datasets. Gigascience. 2015;4: 7.

5. Gilbert E, Carmi S, Ennis S, Wilson JF, Cavalleri GL. Genomic insights into the population structure and history of the Irish Travellers. Sci Rep. 2017;7: 42187.

6. Delaneau O, Marchini J, Zagury J-F. A linear complexity phasing method for thousands of genomes. Nat Methods. 2011;9: 179–181.

7. Lawson DJ, Hellenthal G, Myers S, Falush D. Inference of Population Structure using Dense Haplotype Data. Copenhaver GP, editor. PLoS Genet. 2012;8: e1002453.

8. Hellenthal G, Busby GBJ, Band G, Wilson JF, Capelli C, Falush D, et al. A genetic atlas of human admixture history. Science. 2014;343: 747–751.

9. Commission E-E, Others. Regions in the European Union. Nomenclature of territorial units for statistics. NUTS 2010/EU-27. Luxemburgo: Publications Office of the European Union; 2011.
